# How nurses use National Early Warning Score and Individual Early Warning Score to support their patient risk assessment practice: A fieldwork study

**DOI:** 10.1111/jan.15547

**Published:** 2022-12-21

**Authors:** Caroline S. Langkjaer, Karin Bundgaard, Gitte Bunkenborg, Pernille B. Nielsen, Kasper K. Iversen, Morten H. Bestle, Dorthe G. Bove

**Affiliations:** ^1^ Department of Emergency Medicine Copenhagen University Hospital – North Zealand Copenhagen Denmark; ^2^ Clinical Nursing Research Unit Aalborg University Hospital Aalborg Denmark; ^3^ Clinic for Neuro‐, Head‐ and Orthopaedic Diseases Aalborg University Hospital Aalborg Denmark; ^4^ Department of Public Health, Nursing, Health Faculty Aarhus University Aarhus C Denmark; ^5^ Department of Anesthesiology Copenhagen University Hospital – Holbaek Holbaek Denmark; ^6^ Department of Regional Health Research University of Southern Denmark Odense C Denmark; ^7^ Department of Cardiology Copenhagen University Hospital – Herlev and Gentofte Copenhagen Denmark; ^8^ Department of Emergency Medicine Copenhagen University Hospital – Herlev and Gentofte Copenhagen Denmark; ^9^ Department of Clinical Medicine University of Copenhagen Copenhagen Denmark; ^10^ Department of Anaesthesia and Intensive Care Copenhagen University Hospital – North Zealand Copenhagen Denmark; ^11^ University College Absalon Centre for Nursing Roskilde Denmark

**Keywords:** decision‐making, early warning score, ethnography, nursing, observation practice, participant observation, qualitative research, risk assessment, track and trigger system

## Abstract

**Aim:**

To explore and describe how the National Early Warning Score (NEWS) and Individual Early Warning Score (I‐EWS) are used and how they support nurses' patient risk assessment practice.

**Design:**

A qualitative observational fieldwork study drawing on ethnographical principles was performed in six hospitals in two regions of Denmark in 2019.

**Methods:**

Data were generated from participant observations and informal interviews with 32 nurses across 15 different wards in the hospitals. A total of 180 h of participant observation was performed. The observations lasted between 1.5 and 8 h and were conducted during day or evening shifts.

**Results:**

NEWS and I‐EWS supported nurses' observations of patients, providing useful knowledge for planning patient care, and prompting critical thinking. However, the risk assessment task was sometimes delegated to less experienced staff members, such as nursing students and healthcare assistants. The Early Warning Score (EWS) systems were often adapted by nurses according to contextual aspects, such as the culture of the speciality in which the nurses worked and their levels of competency. In some situations, I‐EWS had the effect of enhancing nurse autonomy and responsibility for decision‐making in relation to patient care.

**Conclusions:**

EWS systems support nurses' patient risk assessment practice, providing useful information. I‐EWS makes it easier to factor the heterogeneity of patients and the clinical situation into the risk assessments. The delegation of risk assessment to other, less experienced staff members pose a risk to patient safety, which needs to be addressed in the ongoing debate regarding the shortage of nurses.

**Impact:**

The findings of this study can help ward nurses, hospital managers and policymakers to develop and improve strategies for improved person‐centred nursing care.

## 1 INTRODUCTION

The National Early Warning Score (NEWS) is a track‐and‐trigger system where patients' vital signs are measured a minimum of twice a day and an aggregated score from 0 to 20 is calculated. Depending on the value of the aggregated score (for which a high score indicates patient deterioration), an escalation protocol dictates the patient monitoring interval, as well as actions to be taken for preventing patient deterioration (Royal College of Physicians, 2017). The NEWS provides a score for risk of deterioration for use in the patient risk assessment. Early Warning Score (EWS) systems are part of nurses' patient risk assessment practice, which includes risk assessment tasks and the nurses' clinical judgement. EWS systems can contribute useful knowledge about the patients' physiological condition and risk of deterioration. Thus, the detection of deterioration is complex, and nurses' clinical judgement is an essential part of the risk assessment of patients. However, Langkjaer et al. (2021) found that neither nurses' clinical judgement nor EWS systems are by themselves sufficient for identifying deteriorating patients.

## 2 BACKGROUND

Since 2012, the NEWS has formed part of nurses' patient risk assessment practice in the eastern part of Denmark. Before the implementation of the NEWS, a study estimated the incidence, staff awareness and subsequent mortality of patients with abnormal vital signs on general wards and found that one out of five patients developed abnormal vital signs during hospitalization and that these patients had a threefold‐increased 30‐day mortality. However, for half of the patients, the nurses were not aware of the presence of abnormal vital signs (Fuhrmann et al., 2008).

The NEWS was implemented to standardize clinical monitoring and to serve as an aid to nurses' clinical assessment as well as decision‐making (Royal College of Physicians, 2017). Several studies have investigated nurses' perceptions of and reactions to the implementation of EWS systems. One study revealed tensions between using a standardized system and relying on clinical judgement, as well as tensions related to rules and compliance (Jensen et al., 2019a). Furthermore, clinical relevance and meaningfulness were identified as being crucial to the implementation process and high compliance with the system (Bunkenborg et al., 2016). While EWS systems have been widely adopted in healthcare systems all over the world, issues involved in the timely recognition of and response to patient deterioration remain complex (Jensen et al., 2018). Patient safety relies to some extent on nurses having the appropriate knowledge and skills for early recognition, reporting and response to patient deterioration, and adverse events may be avoided if these competencies are present. NEWS provides nurses with quantitative knowledge of the patients' clinical condition, but several studies have found a risk of nurses' and physicians' over‐reliance on NEWS and an underestimation of nurses' clinical assessment and intuition (Grant, 2019; Jensen et al., 2018). The underestimation of nurses' clinical assessment and intuition may impact nurses' patient risk assessment practice and be one of the reasons why poor compliance with the NEWS has been found (Credland et al., 2018).

A standardized system like NEWS does not differentiate between different types of diseases or the patient's individual physiological response, which is why it has been referred to as a “one‐size‐fits‐all”‐system (Grant, 2018). Therefore, the Individual Early Warning Score (I‐EWS) system was designed in 2018 as an attempt to develop a standardized assessment system that included a clinical assessment. Studies have found that a structured clinical assessment can provide important information in the recognition and reporting of and response to patient deterioration (Hasselbalch et al., 2019; Iversen et al., 2019). I‐EWS is based on NEWS, which means that nurses record patients' vital signs systematically and follow the NEWS escalation protocol for rescoring. The patients are still assigned an aggregated score from 0 to 20, but with I‐EWS, nurses can adjust the score based on their clinical assessment of the individual patient. The score can be modified in the electronic health record with a maximum of −4 or + 6 points. The nurses can continue with the initial score and simply make a modification of 0 (Nielsen et al., 2022). The criteria and rules of such modifications differed between the wards depending on what the ward management had decided. Prior to the implementation of I‐EWS, local research staff, representing all participating wards, attended teaching sessions.

A cluster‐randomized, multicentre study including 90,964 patients has investigated if I‐EWS performs as well as NEWS regarding clinical outcomes and the use of resources (Nielsen et al., 2022). The study found that I‐EWS performed as well as NEWS in terms of all‐cause mortality at 30 days. However, the study also revealed increased 30‐day mortality in surgical patients. Furthermore, a minimal reduction in routine measurements was found with the I‐EWS, which, in the long term, could lead to a reduction of resources spent (Nielsen et al., 2022). Nurses' experiences and perceptions of using I‐EWS and NEWS are important reasons why a focus group study has explored this topic (Langkjaer et al., 2021). This study found that EWS systems are meaningful to nurses, but the detection of deterioration is complex. Nurses' clinical judgement is an essential part of their patient risk assessment practice. However, Langkjaer et al. (2021) argue that neither nurses' clinical judgement nor EWS systems are by themselves sufficient for identifying deteriorating patients; it is the interaction between the two that supports the identification of patient deterioration. Despite the results from these studies, there is still a need to clarify how NEWS and I‐EWS influence and support nurses' patient risk assessment practice. It is therefore important to explore how nurses use the NEWS and I‐EWS and how these systems support their patient risk assessment practice.

## 3 THE STUDY

### 3.1 Aim

The present study aims to explore how nurses use EWS systems with and without the opportunity to adjust the score based on clinical assessment, and how these systems support nurses' patient risk assessment practice.

### 3.2 Design

A qualitative fieldwork study using participant observation was conducted. Participant observation allowed an investigation of nurses' clinical practices while using I‐EWS and NEWS. Furthermore, this research design enabled an exploration of nurses' knowledge and behaviour in a cultural context (Spradley, 1980).

### 3.3 Sample, participants and settings

Purposeful sampling was used to conduct broad and in‐depth observations, and 32 nurses were recruited (Malterud et al., 2016). The nurses were recruited from 15 different wards in six regional and university hospitals in the Capital Region of Denmark and Region Zealand, Denmark. The nurses were recruited through local research staff, and to ensure an adequate sample size, the recruitment was carried out based on ‘information power’ (Malterud et al., 2016). Information power is related to the following: whether the aim of the study is broad or narrow; the specificity of the participants' experiences and knowledge; the established theory used; the quality of dialogue; and the analysis strategy (Malterud et al., 2016). The participant characteristics are shown in Table 1.

**TABLE 1:** Participant characteristics (N = 32)

	*N* (%)
Age (years)	
20–29	20 (62.5)
30–39	7 (21.9)
40–49	1 (3.1)
50–59	4 (12.5)
Gender	
Female	30 (93.7)
Male	2 (6.3)
Experience (years)	
<1	1 (3.12)
1–4	20 (62.5)
5–10	4 (12.5)
11–20	4 (12.5)
>20	3 (9.38)
Departments	
Medical	17 (53.1)
Surgical	9 (28.1)
Emergency	6 (18.8)

### 3.4 Data collection

A total of 180 h of participant observation were conducted from February to August 2019. During participant observation, the researcher engaged with the research setting and the nurses were aware that they were observed (Spradley, 1980). The observations were conducted by the first author, and each nurse was observed one time. The observations lasted between 1.5 and 8 h, during day or evening shifts. Inspired by Spradley (1980), three different forms of observation were used. For the first form, the focus of the observations was descriptive and open to what was seen, heard and observed when the nurses did the NEWS/I‐EWS scoring. For the second, the observations were more structured and focused on aspects and steps related to NEWS/I‐EWS scoring. Finally, for the third form, the observations were selective and focused on the contrasting aspects of NEWS/I‐EWS scoring (Spradley, 1980). The three forms of observation were often used in a dynamic interaction during the same observation. Examples of the focus of observations are illustrated in Table 2. During the observations, informal interviews were conducted with follow‐up questions to help ensure an understanding of what was observed. Field notes were made during the observations and further expansions, as well as transcription, were completed immediately after each observation. The field notes were written as verbatim and concretely as possible (Spradley, 1980).

**TABLE 2:** Focus of observation and types of questions

Kinds of observations	Descriptive observation	Focused observation	Selective observation
Type of questions	Descriptive	Structural	Contrast
Focus	Open	Ethnographic	Narrow
Kind of questions	Where are vital signs measured?	What are the steps in an EWS measurement?	How are the measurements different from each other?

### 3.5 Ethical considerations

Informed consent was obtained from all participants before inclusion. The study was presented to the Regional Ethics Committee, and according to Danish law, no formal approval was needed (J. no. H‐18053090). All data were anonymized and carefully stored in a secure place approved by the Danish Data Protection Agency (J. no. HGH‐2017‐116, I‐suite no. 06030) and in accordance with the General Data Protection Regulation (GDPR).

### 3.6 Data analysis

An ethnographic analysis was performed based on Hammersley and Atkinson (2007). The analysis started with a thorough reading of the field notes and an initial coding. Based on the initial coding, patterns, concepts and categories were identified. Patterns, concepts and categories were then organized and processed until new categories emerged. The analysis was inductive, which allowed findings to emerge from central and substantial themes in the raw data. To secure trustworthiness in the empirical data, descriptions and quotations from the observations are presented in the findings. The analysis was a back‐and‐forth process between the various steps of analysis, and thus not a linear process. Furthermore, there was a dialectic interaction between the gathering of empirical data and the data analysis of this data, that is, analysis began when the first author identified the area of research, before intensifying during participant observations and then progressing to in‐depth analysis after the fieldwork had been completed (Hammersley & Atkinson, 2007). Researcher triangulation was carried out when performing the analysis. The first two authors made the initial analysis, which was then discussed with the third and last authors until an agreement was reached.

### 3.7 Rigour

All phases, from designing the study to analysis and conclusion, were conducted through collaboration and discussion within the research group. Researcher triangulation contributed to the credibility, confirmability and validation of the data collection and analysis because of the different credentials in the author group. All of the authors are either nurses or physicians and have insider knowledge about the field. This can make it easier to gain access to the field, but it can also mean that some elements are taken for granted and so not observed in‐depth (Wadel et al., 2020). The first author, who conducted the observations, is a registered nurse with a master's degree and is currently a PhD student. She was unfamiliar with the wards and different practices where the data collection took place, which allowed the researcher to be open‐minded and reflexive during observations. To prevent behavioural changes among the observed nurses it was emphasized that the first author was a novice researcher interested in learning about the patient risk assessment practice. Field notes were written as verbatim and concretely as possible to maintain transparency in the observations. Furthermore, the researcher's own reflections were challenged by writing down thoughts and discussing them within the research group.

## 4 FINDINGS

The aim of this study was to explore how nurses use the NEWS and I‐EWS and how these systems support their patient risk assessment practice. Three categories emerged during analysis: (1) Effects on nursing practices, (2) NEWS requires local adaptations and (3) I‐EWS affects nurses' professionalism.

### 4.1 Effects on nursing practices

It was observed that nurses started their shift by collecting information from the electronic health record to gain an overview of the patients he/she was appointed. The nurses collected different information about the patient, including the current NEWS score, former scores and time points for the next risk assessment, as dictated by the escalation protocol. All this information was noted on a piece of paper, which the nurse kept in his/her pocket:

The nurse starts her shift by getting an overview of her patients via the electronic health record. She looks at blood samples, medications, diagnoses and the current NEWS score, former scores, and when the next risk assessment is scheduled. She writes this down on a piece of paper, which she keeps in her pocket. (Fieldnote no. 8, Department of Pulmonary Diseases)

Information about the NEWS score, former scores and when the next risk assessment was scheduled seemed to guide the nurses in planning nursing care for the individual patient. As an example, based on the electronic health record, a nurse noted that she had to test the patient's need for oxygen supply to determine if the patient was ready for discharge. However, the nurse expressed that even though this overview provided information on the patient's status, it had to be validated by seeing the patient:

The nurse says that the electronic health record does not really say anything. I have to go out and see my patient to form a real impression of how they are. (Nurse no. 3, Department of Emergency Medicine)

This validation would often happen when the nurse measured the vital signs, since it created an opportunity to gain knowledge, not only about the physiological condition but also about other relevant aspects. As the following observation emphasized, while measuring the blood pressure, information on how the patient was feeling, how he had slept and his level of pain were explored:

While the blood pressure is being measured, the nurse asks how the patient is doing after his surgery. The patient says that he has slept badly and is in pain. The nurse, therefore, offers the patient extra painkillers. (Fieldnote no. 6, Department of Orthopaedic Surgery)

A nurse expressed that the NEWS was beneficial because the system and the escalation protocol created a framework that guided the risk assessment of the patients. However, a delegation of the risk assessment to less experienced staff members, such as nursing students or healthcare assistants, was observed when the nurses were busy. In some wards, it also seemed to be an integrated part of the culture that nursing students or healthcare assistants were appointed to this task:

A nursing student is at work, and there is a note on which the nurses can write assignments for the nursing student. The nurse writes the next risk assessment of one of his patients on the note, and then the nursing student is going to measure the vital signs instead of the nurse. (Fieldnote no. 2, Department of Emergency Medicine)

During the observations, it became clear that using NEWS was generally perceived by the nurses as being of benefit; the system contributed useful knowledge about the patients' overall physiological condition and risk of deterioration, which the nurses used to plan their nursing care. However, when the risk assessment was delegated to nursing students or healthcare assistants, essential information about the patient's condition might be overlooked which could affect the detection of deterioration.

### 4.2 NEWS requires local adaptations

The way in which NEWS was used for structured observation and scoring of patients' vital signs seemed to be affected by culture, context and the nurses' competencies. In some wards, nurses followed the escalation protocol punctually, while in other wards, nurses had specific hours during which they measured the vital signs. Different cultures of using the EWS systems seemed to be reflected in nurses' clinical practices. Some nurses attached higher values to some of the vital signs than others. However, this valuing of specific vital signs was professionally justified and based on the nurses' knowledge of the patients. For example, for patients with haematology diseases, prevention of infections is especially crucial, and therefore extra attention was paid to the score for fever.

The nurse measures a patient's vital signs and asks if he feels like having a fever. The nurse says that they are very aware of fever in this ward in relation to preventing infections. (Fieldnote no. 33, Department of Hematology)

Different strategies were observed for how the nurses customized the NEWS and the escalation protocol according to the context in which they were using them. The nurses tended to measure vital signs when they found them relevant based on their knowledge of the individual patient and his/her situation, and often the nurses measured the vital signs more than the minimum number of scorings dictated by the escalation protocol. This was observed as being used in the morning to gain a status of the patient's condition, and if something about a patient worried the nurse. It was also observed how nurses prioritized other nursing tasks, such as helping patients' toileting, above measuring vital signs. Prioritizing other nursing tasks was based on the nurses' assessments and reflections on the situation since following the escalation protocol punctually did not always make sense:

The nurse uses the scoring system as a working tool, and she is fully aware that she does not always follow the escalation protocol and says that it just does not always make sense to follow it. (Fieldnote no. 3, Department of Emergency Medicine)

The nurses' competencies to act on patient deterioration were seen to depend on which ward they worked in, the culture and the patient group. It was also seen to depend on the individual nurse. If the nurses were used to handling acute patients and situations, they seemed not to fear patients having a high score. This influenced their use of the EWS system in relation to whether they acted on the high score by themselves or if they involved the physician:

Several of the hospitalized patients on the ward have high NEWS scores. When asked, the nurse said that they are used to dealing with acute conditions or situations, and they are not nervous about patient deterioration. (Fieldnote no. 11, Department of Neurology)

Cultural influences, the current context and the nurses' competencies were observed to affect the observation practice. NEWS requires structured observation and does not take these elements into account. These elements play an essential role in the risk assessment of patients, and therefore the nurses needed to customize NEWS to their context.

### 4.3 I‐EWS affects nurses' professionalism

The opportunity to modify the score when using the I‐EWS was observed to have an impact on nurses' patient risk assessment practice and the frequency with which they conducted patient assessments. The nurses expressed that NEWS and the escalation protocol could be rigid and deprive them of their independence. According to the nurses, I‐EWS impacted their patient risk assessment practice, both regarding their sense of autonomy and regarding having an increased responsibility for preventing patient deterioration. The nurses seemed willing to take on the responsibility of adjusting the score based on their extensive knowledge of the patients, regardless of their level of experience:

The nurse has experienced that I‐EWS gives freedom and increased responsibility. It is a responsibility that she wants to take, as she is the one being with the patients all the time compared to the physicians. (Nurse no. 3, Department of Emergency Medicine)

I‐EWS seemed to trigger critical thinking among nurses, due to the simple fact that it was possible to adjust the score. This critical thinking meant that the nurses paid attention to the single parameters and not only the aggregated score. Hence, the opportunity to adjust gave the nurses an easy and safe way to signal their concerns to both nursing colleagues and to the physician:

The nurse adjusts the score upwards and states that it is an easy and simple way to signal to colleagues and to the physician that she is worried about the patient and that they need to be extra attentive. (Nurse no. 24, Department of Surgery)

During the observation of nurses using I‐EWS, it became clear that there still was a close collaboration between nurses and physicians in relation to the patient's condition. However, it seemed like the nurses' reflections and the opportunity to adjust the score had strengthened their decision‐making and how often they involved the physician. They did not always follow what the escalation protocol dictated, but based their decisions on their clinical assessment of the patient and the situation:

According to the escalation protocol, the nurse should contact the physician, but the nurse chooses to lower the score by ‐2 due to the patient's chronic illness. The nurse has experienced that I‐EWS has reduced unnecessary calls to the physician. She is very pleased about this, as many of the calls were a waste of resources, and the nurse was frustrated that she had to call, even though she had assessed something else. (Fieldnote no. 18, Department of Gastromedical)

On some wards, no adjustments in scores were made, and I‐EWS did not seem to influence the nurses' patient risk assessment practice at all. The nurses expressed that their decisions not to adjust scores were made based on worries of overlooking warning signs if they downgraded the score and not wanting to accept such a responsibility:

The nurse does not want to accept such a responsibility, as he is worried that he overlooks something. The nurse fears that if he adjusts the score and the patient has to be scored later than what the escalation protocol dictates and the patient's condition have worsened, then the nurse would be responsible. (Nurse no. 5, Department of Orthopaedic Surgery)

Some nurses expressed that NEWS and structured observations could support their risk assessment of the patients, albeit most of the nurses stated that the opportunity to adjust the score with I‐EWS made it possible to include their clinical assessment. This opportunity seemed to strengthen their professionalism and make it easier for them to factor the heterogeneity of patients and the clinical situation into their risk assessments.

## 5 DISCUSSION

One main finding of this qualitative observational fieldwork study is that the EWS systems NEWS and I‐EWS support nurses' patient risk assessment practice. The introduction of NEWS means that the nurse or another colleague sees the patients at fixed time intervals, which was not necessarily the case before. Nurses gain knowledge about the physiological condition and risk of deterioration, but they also get an opportunity to assess and identify other relevant aspects of the patient's condition while measuring vital signs. This provides a base of knowledge on which they can plan and initiate individualized nursing care. This is in line with other studies reporting that NEWS is perceived as being beneficial and that routine measurements of vital signs create an opportunity for nurses to do an in‐depth assessment of the patient (Langkjaer et al., 2021; Mølgaard et al., 2022). Furthermore, focusing on signs and symptoms other than vital signs is valuable because nurses' sense of worry and pattern recognition have been found important in providing information in detecting patient deterioration in early stages (Douw et al., 2016; Romero‐Brufau et al., 2019). Therefore, essential information about the patient's condition, that aids nurses in detecting patient deterioration, risks being lost when delegating the risk assessment to nursing students or healthcare assistants. Several studies have raised concerns regarding delegating the task of risk assessment to less experienced staff members without assurance that they have the competencies needed to identify patient deterioration (Grant, 2019; James et al., 2010). Nursing students and healthcare assistants can take on the role of recognizer and recorder; however, the challenge is that less experienced staff members are often unable to interpret the results. They may not have the training or qualifications to understand the physiology and pathophysiology of the measured vital signs, which can result in the undetected deterioration of patients (Beaumont et al., 2008). Thus, the role of the responder will always be the nurses' responsibility.

The nurses perceive NEWS as beneficial; however, they find it problematic that the EWS system is standardized because the standardization means that culture, context and the individual nurse's competencies are not considered important parts of NEWS. Furthermore, the NEWS is a “one‐size‐fits‐all” system that does not consider the heterogeneity of patients (Grant, 2018). When using the NEWS according to the guidelines, nurses considered it to be rigid. Since the NEWS was first implemented, both nurses and physicians in the study settings have been repeatedly taught to pay attention to high NEWS scores. The value of other data and supplementary knowledge about the patient from nurses' clinical assessments have been less emphasized, resulting in a risk that important signs and symptoms are omitted. The result of this emphasis on NEWS could be an overly task‐oriented culture (Grant, 2019). A task‐oriented culture potentially influences nurses' patient risk assessment practice and may contribute to nurses feeling limited in practising person‐centred care. This is consistent with other studies suggesting that nurses feel responsible for something beyond merely following protocols and reporting their actions and that NEWS should be used alongside the nurses' clinical assessment (Alam et al., 2014; Jensen et al., 2019b).

Using NEWS to risk assess hospitalized patients is a complex process and nurses are faced with a paradox: on one hand, there is this task‐oriented, depersonalized and mechanistic approach pushed forward by control‐of‐care standards, for which practical skill dominates; on the other, however, there is a need for understanding and involving the patient and creating a nurse–patient relationship (Kitson et al., 2014). According to the nurses in this study, I‐EWS gave them autonomy and responsibility for their nursing care, and the opportunity to adjust the score called for them to include their clinical assessment when assessing the patients. This opportunity clearly strengthened their professionalism and made it possible for them to factor the heterogeneity of the patients into their risk assessments. This was in line with a study that found that nurses assessed their patients' conditions more accurately when they incorporated their professionalism, competencies and clinical assessment with NEWS (Jensen et al., 2019a). This study argues that the I‐EWS can bridge the two sides of the paradox and make it possible for nurses to practice person‐centred care while using an EWS system.

This study clearly demonstrates that nurses' use of EWS systems like NEWS and I‐EWS supports their patient risk assessment practice. Furthermore, the study clarifies the importance of nurses actively integrating their clinical assessment of the individual patient with the standardized EWS system when identifying and managing patient deterioration. Furthermore, this study highlighted the risk to patient safety of delegating scoring and clinical assessment to students or less skilled staff; a risk that needs to be addressed in the ongoing debate regarding the nursing shortage.

Altogether, the findings of this study can help not only ward nurses but also hospital managers and policymakers to develop and improve strategies for improved person‐centred nursing care.

## 6 LIMITATIONS

The observations were conducted during day and evening shifts, so nurses' use of NEWS and I‐EWS during night shifts do not form part of this study. However, most of the risk assessments were done during day and evening shifts, and if nurses talked about an experience with the EWS systems during a night shift, this was included in the field notes (Spradley, 1980).

The nurses who were willing to participate in this study may represent nurses with specific perceptions of EWS systems. To achieve diversity among the participants, we used purposeful sampling when recruiting the nurses (Malterud et al., 2016). The recruitment focused on varying levels of experience, age and gender. Furthermore, 32 nurses from 15 different wards in six different hospitals participated, which helped to ensure a broad variance in beliefs, practices and experiences.

No Patient and Public Involvement (PPI) was carried out for this work because the participant observations were done with nurses. In future research, it would be interesting to involve the patient perspective on being risk assessed with I‐EWS and, furthermore, to engage in PPI (e.g., to identify topics and choose the right research question).

## 7 CONCLUSIONS

EWS systems support nurses' patient risk assessment practice, providing useful knowledge about patients' overall physiological condition, risk of deterioration and other relevant aspects when planning nursing care. The opportunity to adjust the score with I‐EWS strengthens the professionalism and makes it easier to factor in the heterogeneity of patients and the clinical situation into the risk assessments. However, risk assessments were sometimes delegated to less experienced staff members, which poses a risk to patient safety and needs to be addressed in the ongoing debate regarding nursing shortages.

## AUTHOR CONTRIBUTIONS

CSL, KB, GB, PBN, KKI, MHB, DGB: Made substantial contributions to conception and design, or acquisition of data, or analysis and interpretation of data. CSL; KB, GB, PBN, KKI, MHB, DGB: Involved in drafting the manuscript or revising it critically for important intellectual content. CSL; KB, GB, PBN, KKI, MHB, DGB: Given final approval of the version to be published. Each author should have participated sufficiently in the work to take public responsibility for appropriate portions of the content. CSL; KB, GB, PBN, KKI, MHB, DGB: Agreed to be accountable for all aspects of the work in ensuring that questions related to the accuracy or integrity of any part of the work are appropriately investigated and resolved.

## FUNDING INFORMATION

The work is supported by research grants from Copenhagen University Hospital, North Zealand and Laerdal Foundation (Grant number 3581). The funders had no role in the conduct of the research.

## CONFLICT OF INTEREST

No conflicts of interest have been declared by the authors.

### PEER REVIEW

The peer review history for this article is available at https://publons.com/publon/10.1111/jan.15547.

## Data Availability

The data that support the findings of this study are available from the corresponding author upon reasonable request. All data are in Danish.
